# A qualitative study on stigma experienced by young adults living with sickle cell disease in Accra, Ghana

**DOI:** 10.1093/inthealth/ihac087

**Published:** 2023-01-03

**Authors:** Lydia O Okoibhole, Bassey Ebenso

**Affiliations:** Institute for Global Health, University College London (UCL), Faculty of Population Health Sciences, WC1N 1EH, London, UK; Nuffield Centre for International Health and Development, University of Leeds, School of Medicine, Leeds Institute of Health Sciences, LS2 9JT, Leeds, UK; Nuffield Centre for International Health and Development, University of Leeds, School of Medicine, Leeds Institute of Health Sciences, LS2 9JT, Leeds, UK

**Keywords:** Ghana, non-communicable disease, qualitative study, sickle cell disease, stigma, young adults

## Abstract

**Background:**

Sickle cell disease (SCD) describes a group of multisystem, genetic and stigmatising blood conditions that are prevalent in sub-Saharan Africa. Health-related stigma is a negative experience or adverse social judgement about a group based on an enduring feature conferred by a particular health problem. Literature shows that stigmatisation is experienced by people with SCD with negative implications on their lives. This study investigated self-reported views and lived experiences of young adults in Accra, Ghana, regarding SCD-related stigma and its impact on their lives.

**Methods:**

Data were collected from 19 males and females with SCD using semi-structured individual interviews and focus group discussions. Transcripts were analysed using Braun and Clark's framework for thematic analysis.

**Results:**

Five themes were identified: exclusion; status loss; SCD misconceptions; internalised stigma; and stigma and health outcomes. Overall, interpersonal and institutional levels of stigma were evident throughout the data with a lack of public education, limited specialist care and religion acting as determinants of SCD-related stigma.

**Conclusions:**

Stigma has detrimental consequences for young adults with SCD. Multilevel stigmatisation of SCD at interpersonal and institutional levels should be addressed through multipronged approaches including increased public education, investment in specialist healthcare and collaboration with socioreligious institutions. Further research is needed to investigate the experiences of young adults in rural Ghana.

## Introduction

Sickle cell disease (SCD) is a genetic condition that is growing in global prevalence, affecting millions of people worldwide.^1^ In the next 30 y, it is expected that 15 million children will be born with SCD, predominantly affecting those in sub-Saharan Africa.^2^ SCD is the most prevalent genetic blood condition in Africa, with three-quarters of all cases occurring in the region.^3^ This figure is expected to rise due to an increasing population and globalisation. In Ghana, approximately 2% of children are born with the condition, although this may be an underrepresentation as there is no national screening programme.^4,5^ SCD occurs when an individual inherits abnormal haemoglobin genes from both parents, causing their red blood cells to become sickle shaped. There are various forms of the condition, the most common being sickle-cell anaemia, of which the homozygous form is the most aggressive.^3^

SCD presents in a multitude of ways, such as recurrent infections, chronic fatigue, jaundice, anaemia and debilitating episodes of pain known as crises. It can also lead to various comorbidities such as strokes, delay growth and development, affect sexual function and impact cognitive responses.^1,3,6,7^ Despite this, SCD is often considered an invisible disability and is poorly understood by the population in some contexts, which has contributed to inequality in care, poor health outcomes, social marginalisation, discrimination, stress and stigmatisation, which this study aims to investigate in Accra, Ghana.

Stigma is considered a very significant influence on health and health outcomes, resulting in an increased burden of illness, delays in seeking treatment and can cause individuals to stop treatment earlier than necessary.^8^ Stigma can also affect social status, impacting relationships with friends, peers, family and the public and can also be internalised by individuals.^9–13^ For the purpose of this study, health stigma is defined as ‘a social process or related personal experience characterised by exclusion, rejection, blame, or devaluation that results from experience or reasonable anticipation of an adverse social judgment about a person or group identified with a particular health problem’.^9^

Although there is literature on SCD and stigma, very little of it focuses on Ghana, despite its burden.^8,11,12^ Of the existing literature, very little of it focuses specifically on young adults aged 18–30 y.^11,12^ It has been reported that this group is particularly vulnerable to stigmatisation, which can affect readiness to seek help, among other things.^13^ Young adults also have additional psychosocial challenges that can impact on everyday activities, socialising, forming relationships and overall well-being, which are important to investigate.^11–13^

To fill the gap in the literature, this study aims to investigate the views and everyday experiences of young adults (aged 18–30 y) living with SCD in relation to its stigma in Accra, Ghana. It also seeks to identify the self-reported impact of SCD-related stigma on the health outcomes of young adults in Accra, Ghana.

## Materials and Methods

### Design

Data were collected during 12 June–18 July 2019. All participants were provided with an information sheet that explained the study, were given time to consider their responses and consent forms to sign. This study adopted a qualitative approach, with a cross-sectional study design to identify stigma experienced by young adults living with SCD. Both focus groups and individual interviews (IDIs) were conducted for triangulation.

### Sampling and study setting

A total of 19 eligible participants diagnosed with SCD were recruited using purposive sampling starting from the age of consent (18 y) through to 30 y old (Table 1). This was to capture experiences around dating and marriage, which are culturally significant and impacted by SCD-related stigma.^14^ This was also to comply with the (the University of Leeds) ethics guidelines. Participants were included in the study if they were diagnosed with SCD, were aged 18–30 y and were not debilitated by acute pain or admitted to hospital at the time of the interview. Both males and females were recruited to account for gendered experiences of living with SCD and stigma.^15^ Participants were recruited in person by the lead researcher (LO) with support from a local civil society organisation (Sickle Life) who had contact details of eligible participants through their support groups. Participants were contacted and invited to the study through telephone calls or in person at Sickle Life events or the Ghana Institute of Clinical Genetics (GICG). All participants in this study happen to attend the GICG, a specialist clinic for those with SCD, but the interviews were conducted in a private office space.

Participant involvement in IDIs or focus groups depended on their availability, time constraints and the context in which they were recruited. All participants involved in IDIs happened to be recruited from the clinic and the focus groups were organised through the Sickle Life contact list.

### Data collection

Data were collected using a combination of semi-structured IDIs and focus group discussions (FGDs). A flexible question guide, which was piloted and adapted when appropriate, was used. Questions were formulated using the Health Stigma and Discrimination framework for guidance.^10^ For consistency, a definition of stigma was read out and explained to all of the participants prior to the interviews starting. In total, there were three focus groups, with three participants in each group; there were 10 IDIs, which were audio-recorded. FGDs lasted from 45 min to 1.5 h and the IDIs took 15–30 min. The first author (LO) collected all the data. All participants when recruited consented to taking part in the interviews in English although translators were available if necessary (but they were not required). A sample interview guide for the IDIs and FGDs can be found in Table 2.

**Table 1.** Participant characteristics

Demographic characteristics	Participants (n=19)
Age, y	
Range	18–30
Mean	24.2
Gender	
Female	11 (57.9%)
Male	8 (42.1%)
Genotype	
SS	11 (57.9%)
SC	8 (42.1%)

**Table 2.** Sample interview guide questions about stigma for patients with SCD

**Interview guide questions with probes**
Focus group interview question guide
1. Can you tell me what you know about SCD? (Probe: How do people get SCD, what are its symptoms?) - How do you think Ghanaians generally view and think of SCD? (Probe: Why do you think they see it this way? How does this make you feel?) 2. Tell me about your experience being a [young person] with SCD in Accra, Ghana. 3. How have your experiences made you feel? - Why do you feel this way? 4. Do you tell people about your SCD diagnosis? - How do you feel about telling people about your diagnosis of SCD? (Probe: When you tell people, how do they react? How does their reaction make you feel?) 5. What are people's attitudes towards you when they know your diagnosis? a. How does it make you feel? b. Why do you believe they have that attitude? 6. If relevant, tell me about a time your diagnosis made you feel you like have been treated differently in your everyday life? a. How did it make you feel?
Individual interview questions
1. Can you tell me what you know about SCD? (Probe: How do people get SCD, what are its symptoms?) 2. When were you diagnosed with SCD? 3. Do you tell people about your SCD diagnosis? 4. How do you feel about telling people about your diagnosis of SCD? (Probe: When you tell people, how do they react? How does their reaction make you feel?) 5. If relevant, tell me about a time you felt like your diagnosis has affected your: a. Decision to seek help when in pain/crises. b. Your treatment by a health professional. 6. As a [female/male], do you think sickle-cell has impacted the following: a. Ability to perform your everyday activities (depending on the participant's occupation)? b. Relationships with friends, family and peers? c. Your education? d. The opportunities you are exposed to? e. Mental well-being? f. Psychological well-being?

### Data analysis

Interviews were transcribed verbatim independently by the lead researcher (LO). Using Braun and Clark's^16^ framework for thematic analysis (Table 3), an inductive iterative process was adopted to find the meaning of the data. The data were manually analysed by coding and key themes derived from the data were identified. As themes were identified, relevant quotes were highlighted to use in the article.

**Table 3.** Phases of thematic analysis (Braun and Clarke, 2006)

*Phases of thematic analysis*	*Steps*
Familiarisation with the data	Transcripts were read multiple times for familiarity
Coding	Codes that captured interesting data points were written on transcripts
Search for themes	Codes were combined and grouped together, identifying potential themes
Reviewing themes	Themes were reviewed and developed, with relevant text applied to each theme
Defining and naming themes	Themes (and subthemes) were named and identified
Writing up	Themes were analysed and discussed, with supporting existing literature

## Results

**Figure 1.**
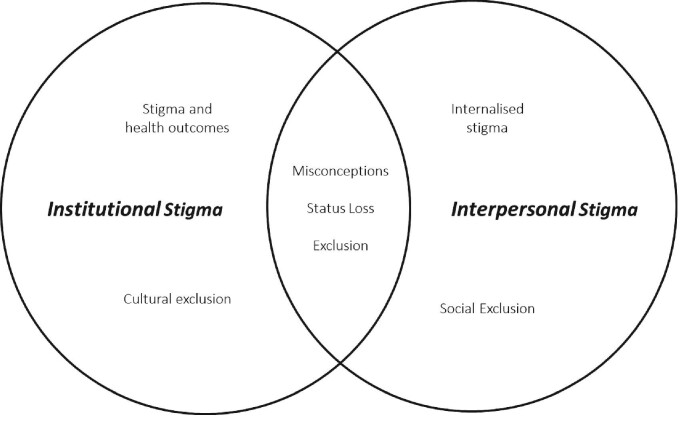
Themes identified under institutional and interpersonal domains. Stigma experienced by young adults in Accra, Ghana. Two broad domains were identified in the data: ‘Institutional’ and ‘Interpersonal’ stigma. Some themes overlapped both institutional and interpersonal stigma, namely, societal ‘misconceptions’ of SCD, ‘status loss’ and ‘exclusion’ of youth living with SCD. ‘Internalised stigma’ can be found under interpersonal and ‘stigma and health outcomes’ under institutional stigma.

Multilevel, interpersonal and institutional sources of stigma were found from the data. Five key themes were identified: ‘exclusion’, ‘status loss’, ‘SCD misconceptions, ‘internalised stigma’ and ‘stigma and health-outcomes’ (Figure 1). All 19 participants gave examples of stigma when we explored their perceptions and lived experiences with SCD stigma, despite the differences in age, gender and genotype.

### Exclusion

Our analysis found that all participants revealed feeling excluded by others for various reasons, which clustered around two subthemes: social exclusion and cultural exclusion.

#### Social exclusion

All participants provided examples of how they were excluded from social activities due to SCD, although the level of exclusion varied. This included not being invited to take part in social activities or events their peers would attend. Participants suggested social exclusion was largely due to societal misconceptions, with many believing those with SCD get sick often and cannot get involved in certain activities. However, individuals also described being excluded by friends and family, people who often had a good understanding of their condition. However, it was also suggested that many friends and families believed those with SCD exaggerate their symptoms and this may be another reason for this exclusion.

*My friends would go like, because I don't want to go on their trip, I'm faking… yes, I'm faking pain, I'm faking sick…. They don't involve me when they want to do something or go out or have fun* (Female, IDI.3).

Several participants discussed being called a ‘Sickler’, a term which had very negative connotations and was interpreted as a way of othering those with SCD, making them feel different or incapable. When asked how it made them feel, many described not feeling ‘normal’, feeling like an ‘outsider’ and being ‘put into a box’.

*I don't know why; they just attach that label. I am not a Sickler. That's what annoys me, if someone calls me a Sickler we will probably have a fight at that point* (Male, FGD.1.3).

#### Cultural exclusion

When asked about stigma and its impact on their lives and relationships, many described the importance of dating, marriage and children in Ghanaian tradition. In a country like Ghana, marriage is a central social institution that enables partners to satisfy their physical, psychological, social, cultural and economic needs and to form bidirectional relationships, leading to a stable family.^17^ Marriage was the main cultural aspect of stigma cited by participants and the overall perception was negative. Many described being interested in the idea of dating and marriage but mentioned ‘being rejected’ by prospective partners, due to their diagnosis, which may have contributed to all participants insisting that anyone they marry must have the AA genotype, due to wanting to have children that were not diagnosed with SCD. One participant described not marrying for love to ensure that her children did not have SCD. Another described potential partners and their families rejecting her due to her status.

*And so do other people. Even men. I'm still not married. I am dating but whenever I disclose my genotype they change, or their families say no. It's hard* (Female, IDI.8).

### Status loss

All participants described feeling like their diagnosis affected their social status and, in turn, limited opportunities they were given or exposed to. This was often tied to misconceptions around the condition and negative stereotypes.

One participant gave an example of being discriminated against in the workplace due to SCD that resulted in social loss of status that subsequently affected their ability to progress. It also led to feelings of personal disappointment and dissatisfaction.

*Yes definitely, all the time. Like my current job, I am due a promotion. I want the promotion; I have worked so hard for it! But my bosses told me that they can't give it to me because I have sickle cell* (Female, IDI.8).

When probed about this, the participant described this was expected, given the high prevalence of institutional stigma that exists in Ghana around SCD, despite this action being illegal based on Ghana's disability laws.^18^ Comparisons were also made with other conditions, and it was clear that the stigma associated with SCD was very high and much more overt.

*…this is Ghana, we do not have such things especially in the private sector. I am just thinking of moving or just working hard to prove I can do it. They know I can. And how does it make me feel did you say? Erm, bad. Very bad. I am normal, I don't feel like I deserve to be treated this way. It's like my cousin, she has asthma or someone with diabetes. It's something you just live with. But with sickle cell, the stigma is so high, people treat you so differently when they hear you have it* (Female, IDI.8).

Another participant, who captured her experience of institutional stigma, mentioned being told by a university teacher to drop out of her degree because of her diagnosis, despite her doing well. Participants felt that their SCD diagnosis made them be seen as disabled, something that has social and personal implications in Ghana^18^ and targeted them as ‘other’, resulting in a loss of status.

### Misconceptions of SCD

Misconceptions of SCD, how it is spread and its effect on the body, are what many participants argued contributed to the stigmatisation of SCD. When asked about the perceptions of SCD from society, some said people expected them to look a certain way and mentioned being told that they would ‘die early’ due to SCD. When asked why they believed they were seen this way, all participants agreed that most Ghanaians had a poor understanding and misconceptions of SCD. However, the majority recognised that public awareness has improved in recent years.

These misconceptions include how people get SCD, which is an inherited, genetic condition. All participants were aware of this but described being told a range of inaccurate facts about the causation of SCD, including it being ‘transmitted’ by coughing and sneezing. Some mentioned they were told that it was a ‘curse’ or a ‘spiritual attack’.

*There's some who think it's an airborne thing. That's what some people think, I don't know why. They think it's something so… they associate it with something very very very dangerous… So, they tend to look at you in a bad way* (Female, IDI 9).

*One thing is the culture. People still think that sickle cell disease is a curse. If you have a child with it, your child is evil* (Female, FGD 3.1).

Several participants acknowledged that misconceptions contributed to the stigma faced by individuals but argued that those views were dying out due to increased awareness and were largely reserved for those living in rural areas, not Accra, where this study was set.

Another misconception was around the treatment for SCD. All participants described ways to manage SCD but recognised it cannot be cured. However, several participants mentioned that they had been told it can be cured through prayer. The majority of participants described themselves as being religious and found this claim ‘hurtful’ and ‘confusing’. Some also felt like their faith was being challenged if their SCD was not cured. One participant described being isolated from family because they stopped going to prayer camps, centres run by religious institutions where people can seek healing from their ailments through prayer.^19^

*That was a confusing time for me, you've heard all these things growing up, medical things, so it was hard to hear that they can cure you by praying… if you refuse to go to a prayer camp, they will see you in a certain light. As if you don't believe in God* (Male, FGD 1.3).

However, several participants made reference to the role of the church in ‘pairing couples’ based on their genotype, whereby they can prevent those carrying the SCD trait from marrying anyone who also has it,^14^ suggesting that religion is very influential. This misconception and practice overlaps with cultural exclusion as a subtheme, under which the cultural institution of marriage was discussed.

### Internalised stigma

Internalised stigma is when individuals accept stigmatising assumptions about their condition. This theme was interpreted based on many participants mentioning that they do not disclose their condition to others, unless essential. This was due to not wanting to be judged or treated differently. Many also used the word ‘normal’ when describing how they wanted to be and live. When asked why, they argued that society does not see SCD or those living with it as ‘normal’, and they do not want to be treated that way. This view was mostly held by women, particularly those aged <24 y, which may indicate a gendered difference. One participant compared SCD with HIV:

*When you tell someone you have HIV… automatically, people change towards you… also when I tell someone [I have SCD], I feel like the person will change towards me. Some of them do* (Female, IDI.4).

Those who avoided telling others about their condition described a feeling of ‘fear’ or being ‘nervous’ at the prospect of telling people. However, it is important to acknowledge that for some, they did not disclose because they thought SCD was a personal issue they wanted to keep to themselves. The minority who were open about telling people acknowledged that it was easier as they became older and more accepting of their diagnosis.

### Stigma and health outcomes

This theme addresses the correlation between stigma and poor self-reported health outcomes.^11^ When asked if their diagnosis has affected their decision to ‘seek help when in pain/crises’, there were varying responses. Many said sickle cell crisis did not affect seeking help, as they felt comfortable telling those close to them, but some participants said it does, due to not wanting to feel like a ‘burden’ to their family and friends.

*Yeah, sometimes I have a crisis, but I normally don't like people getting to know… instead of sympathising with you, they pity you… I decided to not tell people because I don't want people to pity me. Because you can sympathise with someone, that means you care but to pity the person, it's like diminishing the person* (Female, IDI.5).

When asked about their mental well-being, many participants described feeling ‘depressed’, particularly when in pain, intentionally isolating themselves from others. One participant described having suicidal thoughts due to a crisis, which resulted in vascular necrosis.

*I had suicidal thoughts at some point. When the vascular necrosis started, I was in so much pain. I was scheduled for surgery a year later, but I was in so much pain that I had to use crutches until then. After the surgery, I had to use it for another 1–2 years and I got so depressed and felt suicidal* (Male, FDG 2.3).

When asked whether SCD-related pain affected their ‘treatment by a health professional’, the responses depended on where individuals were treated, for example, in a specialist clinic or general hospital. All participants were registered and treated at the GICG and descriptions of that clinic were largely positive. However, this changed when participants were treated out of hours or at hospitals without specialist personnel. Several participants suggested that the level of care varied depended on location. In one FGD, all participants agreed that healthcare in Ghana was ‘inequitable’ due to unequal funding, with the best care available in Accra, the capital. This may affect the levels of stigma experienced by people in different areas of the country.

## Discussion

There is growing interest in researching the perspectives of young adults with SCD in Ghana,^20–23^ many of which focus on transition care rather than on SCD-related stigma. This study is the first to investigate stigma experienced by young adults living with SCD in Accra, Ghana. Other studies have focused on different regions in Ghana.^23^ The five themes that occurred from the analysis indicate that for young people in this context, health-related stigma influences their experiences as persons with SCD in Ghana. Overall, the sources of stigmatisation were multilevel, with interpersonal and institutional stigma identified throughout the data. Considering this, all strategies to tackle stigma surrounding SCD must be specific to the context and adopt a multipronged approach.

Institutional stigma involves policies of government and private organisations as well as sociocultural practices that limit opportunities for people with certain conditions.^24^ The institution of marriage within the construct of socioreligious practices^17^ consistently came up in the data as a source of stigma. Culturally, marriage and having children are seen as a rite of passage, particularly for women.^15^ Marriage also ties in with religion, which is also a huge influence on stigma in Ghana, where only 1.1% of the population describe themselves as non-religious.^25^ Prayer was often described as a curative option and the church actively play a role in pairing couples with particular genotypes. Religion has been found to have both a positive and negative impact on those with SCD, bringing both emotional respite and being a source of physiological distress,^11,26^ but could be used as an interventional tool in this context and previous studies have adopted this approach.^26^

Other sources of institutional stigma include the health system. Existing studies found that there was stigma among health professionals dealing with SCD patients,^11,13,27^ but in the context of SCD care in Accra, it was positive. All participants in this study attended the GICG, a specialist clinic for those with SCD. Having specialist care made a difference to many of their experiences, so working with health professionals could play a role in reducing the stigmatisation of SCD. Those who recalled not being treated well were treated outside of the GICG. In the case of HIV and other medical conditions, specialist care has successfully been used to tackle the burden and reduce health system stigmatisation.^28^

On the other hand, interpersonal stigma involves discrimination from peers, friends and family,^24^ leading to those living with SCD feeling judged, devalued and mistreated, particularly in social circles, and this is significant for young adults who are still developing and facing life-changing experiences. This can be hugely detrimental and can go on to impact other aspects of their lives such as relationships and job opportunities. This may also cause those with SCD to be more private about their condition, as a means of fitting in, to avoid potentially putting themselves at risk if they disclose their condition.^13^

The WHO has recognised the role of peers in helping to tackle health stigmatisation, citing ‘knowledge is power’ and encouraging ‘social contact’ with those who have a health condition, as a means to improve public education of stigmatised health conditions.^28^ Peer-led interventions have previously been found to be successful^29^ on an interpersonal level as they improve public awareness, but have also been found to reduce self-stigma and increase individual empowerment.^30^ In the context of this study, this could be successful. All participants reported that public knowledge could be improved in the context of prevailing misconceptions leading to further stigmatisation, despite good intentions from family and friends.

Considering this, interventions to reduce SCD-related stigma against young adults in Accra would benefit from collaborations with religious institutions and schools to reduce stigma, investment in the health system and working with government departments such as the Ministry of Information to promote health education about SCD for the general population. Studies conducted in similar settings have called for a similar approach to be taken.^23,26^

### Limitations

Data collection was conducted during the rainy season in Ghana and the cold often triggers a crisis for those with SCD.^1^ Overall, this did not prevent themes to be identified. Data were also coded and analysed by one person, so the views articulated in this study are through the lens of one person's knowledge and experience.

Many participants did not originally understand the word ‘stigma’, which may have been due to it not often being used in a Ghanaian context. To overcome this, for consistency, the same definition of health-related stigma was provided to each participant and asked if it was understood before interviews were conducted. This also contributed to rigour.

### Conclusions

The importance of stigmatisation in the lives of people living with SCD is increasingly being recognised although studies remain relatively few in this area. SCD-related stigma is prevalent in Ghana and has detrimental consequences for young adults with the condition, affecting individuals physiologically and psychologically.

Based on the data analysis and discussions with sickle-cell advocates, health professionals and those with the condition, we recommend that measures to tackle interpersonal and institutional stigma are taken. Collaboration with socioreligious institutions, public education and improved public awareness should be promoted and further investment should also be made into specialist SCD care. Further research on this topic is recommended, particularly research on the experiences of young adults in rural regions, as many misconceptions of SCD were described by participants and in previous studies as being from people in those areas.^23,26^

## Data Availability

The data underlying this article will be shared on reasonable request to the corresponding author.
